# 基于真菌毒素污染差异的液相色谱-串联质谱法鉴别板栗粉中掺假小麦粉

**DOI:** 10.3724/SP.J.1123.2021.10021

**Published:** 2022-04-08

**Authors:** Jian ZHOU, Xiaohong CHEN, Micong JIN

**Affiliations:** 宁波市疾病预防控制中心, 浙江省微量有毒化学物健康风险评估技术研究重点实验室, 浙江 宁波 315010; Ningbo Municipal Center for Disease Control and Prevention, Key Laboratory of Health Risk Appraisal for Trace Toxic Chemicals of Zhejiang Province, Ningbo 315010, China; 宁波市疾病预防控制中心, 浙江省微量有毒化学物健康风险评估技术研究重点实验室, 浙江 宁波 315010; Ningbo Municipal Center for Disease Control and Prevention, Key Laboratory of Health Risk Appraisal for Trace Toxic Chemicals of Zhejiang Province, Ningbo 315010, China; 宁波市疾病预防控制中心, 浙江省微量有毒化学物健康风险评估技术研究重点实验室, 浙江 宁波 315010; Ningbo Municipal Center for Disease Control and Prevention, Key Laboratory of Health Risk Appraisal for Trace Toxic Chemicals of Zhejiang Province, Ningbo 315010, China

**Keywords:** 分散固相萃取, 超快速液相色谱, 串联质谱, 真菌毒素, 板栗粉, 小麦粉, 掺假, 鉴定, dispersive solid-phase extraction (d-SPE), ultrafast liquid chromatography (UFLC), tandem mass spectrometry (MS/MS), mycotoxin, chestnut flour, wheat flour, adulteration, identification

## Abstract

建立了分散固相萃取-超快速液相色谱-串联质谱法同时测定板栗粉和小麦粉中43种真菌毒素的方法,对48份板栗粉和80份小麦粉样品的污染状况进行调查,筛选出5种专属于小麦粉的标志性真菌毒素。样品采用84%(v/v)乙腈水溶液提取,提取液采用C_18_结合增强型脂质去除净化剂(EMR-Lipid)净化,采用响应曲面-中心组合设计优化分散固相萃取净化方法。净化液在BEH C_18_色谱柱(100 mm×2.1 mm, 1.7 μm)上分别采用0.1%甲酸水溶液和含0.1%甲酸的甲醇-乙腈(1:1, v/v)(电喷雾正离子模式)、水和乙腈(电喷雾负离子模式)为流动相进行梯度洗脱,分别采用电喷雾电离(ESI)正负离子模式检测,基质匹配曲线外标法定量。板栗粉中真菌毒素的3水平加标回收率在72.4%~109.4%之间,相对标准偏差(RSD)<7.5%;小麦粉中真菌毒素的3水平加标回收率在70.7%~112.9%之间,RSD<9.3%;两种基质中43种真菌毒素的定量限均在0.1~20.0 μg/kg之间,方法线性相关系数均大于0.9991。利用所建立的方法监测了128份样品,结果表明,两种基质普遍受到真菌毒素污染,其中脱氧雪腐镰刀菌烯醇及其衍生物3-乙酰化-脱氧雪腐镰刀菌烯醇、15-乙酰化-脱氧雪腐镰刀菌烯醇、雪腐镰刀菌烯醇、去环氧-脱氧雪腐镰刀菌烯醇仅在小麦粉中检出。采用GB 5009.111-2016同位素稀释液相色谱-串联质谱法验证,检测结果与本方法一致。所建立的方法简便、快速、灵敏、准确,可有效满足板栗粉和小麦粉中真菌毒素残留的检测要求,脱氧雪腐镰刀菌烯醇及其4种衍生物可以作为两种食品的掺假标志物。

## Abstract (en)

An analytical method based on dispersive solid-phase extraction (d-SPE) and ultrafast liquid chromatography-tandem mass spectrometry (UFLC-MS/MS) was employed for the determination of 43 mycotoxins in chestnut flour and wheat flour. A total of 128 samples consisting of 48 chestnut samples and 80 wheat flour samples were collected randomly and subjected to analysis. Finally, five specific toxins were selected as markers to identify these two foodstuffs. Acetonitrile-water (84:16, v/v) was used to extract mycotoxins from chestnut flour and wheat flour. After extraction, the supernatant was transferred to the d-SPE equipment, using which purification was performed with C_18_ and EMR-Lipid (lipid adsorbent). Chromatographic separation was carried out by gradient elution with eluent A (ESI^+^: 0.1% formic acid, ESI^-^: water) and eluent B (ESI^+^: methanol-acetonitrile (1:1) containing 0.1% formic acid, ESI^-^: acetonitrile) on a BEH C_18_ column (100 mm×2.1 mm, 1.7 μm). Quantitative analysis was performed with the aid of matrix-matched curves. When establishing the method, the experimental matrix for optimization was designed by central-composite design based on the response surface methodology. Quadratic polynomial equations were deduced to describe the relationships between the responses and variables, and assess the interaction effects among the variables to acquire the true optimal conditions with less workload. Using the optimum experimental conditions, the accuracy of the proposed method was determined through three-level spiking tests, while the precision was evaluated in terms of the repeatability (six replications per level). Satisfactory precisions (RSDs≤7.5% in chestnut flour and RSDs≤9.3% in wheat flour) were achieved in all tested assays. The recoveries were also acceptable, and ranged from 72.4% to 109.4% for chestnut flour and from 70.7% to 112.9% for wheat flour. The matrix effects of mycotoxins were 48%-128% in wheat flour and 41%-112% in chestnut flour. The detectability of mycotoxins in the two matrices was assessed by spiking the blank extracts with various low concentrations, and determined as the lowest values that can produce chromatographic peaks at a signal-to-noise ratio (*S/N*) of 3:1. The obtained limits of quantification varied from 0.10 μg/kg to 20 μg/kg (bongkrekic acid) in both investigated matrices. Satisfactory linearities were obtained, with correlation coefficients>0.9991 for all the analytes. After validation, the contamination status of the multiple mycotoxins was evaluated for various concentration ranges. Based on the obtained data, both wheat flour and chestnut flour were severely contaminated, with 17 mycotoxins detected in them. Particularly, chaetoglobosin A, ochratoxin B, and penicillic acid were only detected in chestnut flour, while 3-acetyl-deoxynivalenol, deoxynivalenol, and nivalenol were detected in wheat flour. Further, the positive rates and contamination concentrations of chaetoglobosin A, ochratoxin B, and penicillic acid were not significant; hence, they did not qualify as identification markers. On the other hand, the incidence of deoxynivalenol in wheat flour almost reached 100%, which is very significant. Finally, deoxynivalenol and its four derivatives (3-acetyl-deoxynivalenol, 15-acetyl-deoxynivalenol, deepoxy-deoxynivalenol, and nivalenol) were treated as adulteration markers for the two foodstuffs. To improve the reliability of the conclusion, all samples were re-tested using the first method prescribed by the National Food Safety Standard, i. e., GB 5009.111-2016. Ten chestnut flour samples were also randomly selected to prepare moldy samples under suitable environmental conditions for the growth of *Fusarium*, to verify the production and release of deoxynivalenol and its derivative mycotoxins under the extreme conditions. The distribution data for these mycotoxins were consistent with those obtained by d-SPE, confirming that the adulteration criterion is trustworthy.

The established method is simple, rapid, sensitive, and accurate, and can effectively meet the requirements for the simultaneous determination of multiple mycotoxins in chestnut flour and wheat flour. Moreover, the adulteration results, which were obtained for natural contaminants (deoxynivalenol and its four derivatives), are less affected by humans and hence, much more accurate and reliable.

板栗(*Castanea mollissima Blume*)是我国的重要经济作物,种植历史超过3千年,素有“铁杆庄稼”之美称^[1,2]^。目前,我国已成为世界上最大的板栗生产国和消费国,2019年产量超过227万吨^[3]^。板栗采收期通常集中在每年的9-10月份,收获季气温较高且新鲜板栗含水、含糖量高,不耐贮藏,极大地限制了板栗产业的发展^[4]^。利用干燥技术脱水制成板栗粉可大大延长保质期。板栗粉中的膳食纤维、多酚、黄酮类物质均显著高于小麦粉,且具有无麸质的优点^[5,6]^。通常情况下,板栗粉与小麦粉市场价格相差5~7倍,巨大的成本差异促使部分不法商贩混合售卖,单凭肉眼和口感很难区别。目前植源性食品的真实性鉴定方法主要包括稳定同位素质谱法、近红外分析法、紫外-可见分光光度法等^[7,8,9]^,也有基于蛋白质、淀粉含量差异的粗略定性技术^[10]^。其中,稳定同位素质谱法测定准确度较高,但所需设备价格昂贵且用途单一;近红外分析法、紫外-可见分光光度法的测定准确度和灵敏度均不如同位素质谱法,且易受空白标样的影响。因此开发一种快速、有效、准确的板栗粉中掺假小麦粉的鉴定方法具有重要的实际意义。

真菌毒素是一类由产毒真菌(以青霉属、曲霉属、镰刀菌属和链格孢霉属为主)产生并释放的有毒次级代谢产物^[11,12]^,具有强烈的“三致”作用和细胞毒性^[4,13]^。作物通常在种植过程中就受到真菌侵染,人为干预仅能控制食物中部分真菌毒素的浓度,而很难将毒素彻底去除,因此将特定的真菌毒素作为掺假标志物对于开发食物新型掺假鉴定技术具有很强的参考价值。

目前针对板栗粉的研究主要集中在产毒菌种的分离和鉴定方面,如曲霉、青霉、链格孢霉等^[14,15]^,关于板栗和面粉中多真菌毒素污染的研究也比较有限,通常仅监测如黄曲霉毒素、赭曲霉毒素等重点毒素^[15,16,17,18]^。因此,开发建立板栗粉和面粉中真菌毒素的多组分同时分析方法并研究其污染分布特征具有非常重要的现实意义^[3]^。目前适用于多真菌毒素的预处理方法主要包括固相萃取(SPE)、稀释进样法、多功能净化柱法、分散固相萃取法等^[12]^。其中,稀释进样法对仪器灵敏度要求极高,且存在回收率低、重现性较差等问题,同时未净化样液直接进样会大大增加色谱柱和离子源污染的可能性^[19]^;多功能净化柱虽然处理速度最快,但对复杂基质净化效果有限,通常仅适用于单类毒素(如黄曲霉毒素或单端孢霉烯族毒素)的测定^[20]^; SPE技术被广泛应用于真菌毒素测定,但操作相对繁琐耗时、分析成本高,净化效果受基质复杂程度和目标毒素性质限制;分散固相萃取法(dispersive solid-phase extraction, d-SPE)因具有快速、简单、廉价、高效、稳定、安全等特点,近年来逐渐被应用于真菌毒素分析^[21,22,23]^,但在多组分分析方法建立过程中存在多实验变量、多目标响应的情况,如果采用传统控制变量法,则无法评估各因素的重要程度,忽视因素间交互效应,且所需试验次数显著增多^[24,25]^。

本研究拟采用C_18_结合增强型脂质去除净化剂(EMR-Lipid)d-SPE净化技术,首先筛选确定合适的净化剂,后结合响应曲面-中心组合设计实验矩阵对其用量比例进行优化,建立各真菌毒素的二次多项式拟合模型并计算得到理论最佳实验条件。最终建立准确、灵敏的板栗粉和小麦粉中43种真菌毒素的d-SPE-超快速液相色谱-串联质谱(ultrafast liquid chromatography-tandem mass spectrometry, UFLC-MS/MS)测定方法,并应用于板栗粉中掺假小麦粉的鉴定。

## 1 实验部分

### 1.1 仪器、试剂与材料

ExionLC液相色谱仪(日本Shimadzu岛津公司); AB Sciex Q-Trap 6500 plus三重四极杆质谱仪(美国AB Sciex公司); Milli-Q超纯水仪(美国Merck公司)。

乙腈、甲醇、甲酸(色谱纯)购自赛默飞世尔科技(中国)有限公司;试验用水为Milli-Q制备超纯水;d-SPE净化剂(包括EMR-Lipid、C_18_、PSA(*N*-丙基乙二胺)、GCB(石墨化炭黑)、PCX(混合型阳离子交换)、PAX(混合型阴离子交换)吸附剂)分别购自安捷伦科技有限公司和飞诺美&博纳艾杰尔科技有限公司。

43种真菌毒素固体标准品,纯度均大于98%,具体化合物名称见表1,均购自青岛普瑞邦生物工程有限公司。标准储备液的配制:准确称取1.0 mg标准品于10 mL容量瓶中,用乙腈溶解并定容至刻度,此溶液中真菌毒素的质量浓度为100.0 mg/L,于-20 ℃冰箱中避光保存。真菌毒素标准工作溶液按照ESI^+^和ESI^-^两种模式进行配制:依次取适量真菌毒素标准储备溶液于5 mL容量瓶中,用乙腈稀释至刻度,配成质量浓度如表1所示的两种真菌毒素标准混合工作溶液分别用于ESI^+^和ESI^-^模式测定,其中ESI^+^混合溶液中共包含24种毒素,而ESI^-^混合溶液中共包含19种毒素。同位素内标标准工作溶液:准确移取^13^C_15_-脱氧雪腐镰刀菌烯醇、^13^C_15_-雪腐镰刀菌烯醇、^13^C_17_-3-乙酰基-脱氧雪腐镰刀菌烯醇标准溶液(25 mg/L, Romer公司)用乙腈配制成1.0 mg/L,密封,于-20 ℃条件下避光保存,备用。

**表1:** 43种真菌毒素的MRM参数

Mycotoxin	Mass concentration/ (mg/L)^1)^	ESI polarity	Retention time/min	Precursor ion (m/z)	Quantitative ion (m/z)^2)^	Qualitative ion (m/z)^2)^
Aflatoxin B_1_(黄曲霉毒素B_1_)	0.5	ESI^+^	3.33	313.0	285.0 (30)	241.1 (48)
Aflatoxin B_2_ (黄曲霉毒素B_2_)	0.5	ESI^+^	2.78	315.2	259.0 (38)	287.0 (32)
Aflatoxin G_1_ (黄曲霉毒素G_1_)	0.5	ESI^+^	2.54	329.2	243.2 (35)	283.0 (33)
Aflatoxin G_2_ (黄曲霉毒素G_2_)	0.5	ESI^+^	2.15	331.2	245.2 (38)	285.0 (35)
Aflatoxin M_1_ (黄曲霉毒素M_1_)	0.05	ESI^+^	1.93	329.1	273.0 (33)	301.0 (25)
Altenuene (交链孢烯)	4.0	ESI^+^	4.44	259.2	184.8 (40)	213.2 (36)
Beauvericin (白僵菌素)	0.5	ESI^+^	11.01	784.4	244.2 (34)	262.2 (33)
Chaetoglobosin A (球毛壳菌素A)	4.0	ESI^+^	7.19	529.3	130.1 (50)	511.3 (13)
Diacetoxyscirpenol (蛇形菌素)	2.0	ESI^+^	3.79	384.3	307.0 (15)	247.0 (18)
Enniatin A (恩链孢菌素A)	0.5	ESI^+^	11.87	682.5	210.2 (35)	555.5 (35)
Enniatin A_1_(恩链孢菌素A_1_)	0.5	ESI^+^	11.42	668.5	210.2 (35)	541.4 (37)
Enniatin B (恩链孢菌素B)	0.5	ESI^+^	10.66	640.5	196.2 (32)	527.4 (32)
Enniatin B_1_(恩链孢菌素B_1_)	0.5	ESI^+^	11.05	654.5	196.2 (32)	541.3 (33)
Gliotoxin (胶黏霉素)	5.0	ESI^+^	3.04	327.3	263.1 (13)	227.1 (23)
HT-2 (HT-2毒素)	20.0	ESI^+^	4.83	425.3	215.0 (16)	263.0 (16)
Neosolaniol (新茄病镰刀菌烯醇)	5.0	ESI^+^	1.26	400.3	305.0 (16)	185.0 (25)
Ochratoxin A (赭曲霉毒素A)	0.5	ESI^+^	6.55	404.0	239.0 (32)	358.0 (18)
Ochratoxin B (赭曲霉毒素B)	0.5	ESI^+^	5.21	370.2	205.0 (29)	324.0 (17)
Penicillic acid (青霉酸)	0.4	ESI^+^	1.44	171.1	125.1 (17)	97.1 (22)
Sterigmatocystin (杂色曲霉毒素)	0.5	ESI^+^	6.65	325.1	310.0 (33)	281.0 (48)
T-2 (T-2毒素)	1.0	ESI^+^	5.82	484.1	305.0 (18)	185.0 (25)
Tentoxin (腾毒素)	1.0	ESI^+^	4.91	415.2	312.3 (29)	256.3 (41)
Toxoflavin (毒黄素)	1.0	ESI^+^	0.93	194.0	137.0 (21)	109.0 (27)
Verruculogen (疣孢青霉原)	1.0	ESI^+^	8.09	534.0	392.2 (17)	498.3 (18)
15-Acetyl-deoxynivalenol (15-乙酰化-脱氧雪腐镰刀菌烯醇)	2.0	ESI^-^	3.60	337.1	150.0 (-20)	219.0 (-19)
3-Acetyl-deoxynivalenol (3-乙酰化-脱氧雪腐镰刀菌烯醇)	2.0	ESI^-^	3.69	337.1	307.0 (-15)	173.0 (-15)
Alternariol monomethyl ether (交链孢酚单甲醚)	0.10	ESI^-^	6.15	271.2	256.2 (-29)	228.1 (-35)
α-Zearalanol (α-玉米赤霉醇)	0.12	ESI^-^	5.59	321.0	277.2 (-31)	303.0 (-30)
α-Zearalenol (α-玉米赤霉烯醇)	0.12	ESI^-^	5.66	319.1	275.1 (-28)	301.1 (-28)
Bongkrekic acid (米酵菌酸)	2.0	ESI^-^	11.01	485.3	441.2 (-15)	397.3 (-25)
β-Zearalanol (β-玉米赤霉醇)	0.12	ESI^-^	5.37	321.1	277.0 (-30)	303.0 (-30)
β-Zearalenol (β-玉米赤霉烯醇)	0.12	ESI^-^	5.42	319.1	275.1 (-29)	301.1 (-28)
Citreoviridin (黄绿青霉素)	2.0	ESI^-^	5.80	401.3	300.0 (-23)	285.1 (-33)
Deepoxy-deoxynivalenol (去环氧-脱氧雪腐镰刀菌烯醇)	1.0	ESI^-^	2.84	279.2	249.0 (-12)	231.0 (-20)
Deoxynivalenol (脱氧雪腐镰刀菌烯醇)	5.0	ESI^-^	2.53	295.4	265.1 (-14)	138.0 (-22)
Fumagillin (烟曲霉素)	0.5	ESI^-^	8.01	457.4	131.0 (-25)	413.3 (-19)
Fusarenone X (镰刀菌酮X)	2.0	ESI^-^	2.90	353.2	263.2 (-15)	187.0 (-32)
Nivalenol (雪腐镰刀菌烯醇)	2.0	ESI^-^	1.63	311.0	281.0 (-14)	191.0 (-26)
Patulin (展青霉素)	2.0	ESI^-^	1.87	153.0	81.0 (-16)	108.8 (-11)
Penitrem A (震颤霉素A)	0.5	ESI^-^	8.11	632.2	564.2 (-42)	295.0 (-60)
Wortmannin (渥曼青霉素)	2.0	ESI^-^	5.45	427.1	384.1 (-23)	308.2 (-33)
Zearalanone (玉米赤霉酮)	0.12	ESI^-^	6.10	319.0	275.0 (-28)	205.0 (-32)
Zearalenone (玉米赤霉烯酮)	0.12	ESI^-^	6.19	317.1	174.9 (-31)	130.8 (-27)

1) In two mixed standard solution, respectively. 2) Collision energy (eV) is given in brackets.

板栗粉48份(其中现成板栗粉22份,其余26份为板栗仁、干栗于实验室内研磨、烘干制备而成)和小麦粉80份(购自电商平台及宁波当地超市)。自制霉变板栗粉10份:随机挑选10份板栗粉样品100 g分别平铺于瓷盘上,置于20±5 ℃、70%±10%相对湿度的自然环境条件,以明显气味改变或肉眼可见霉菌为止。

### 1.2 色谱-质谱条件

色谱柱:Waters BEH C_18_柱(100 mm×2.1 mm, 1.7 μm);柱温:40 ℃;流速:0.3 mL/min;进样体积:10 μL; ESI^+^模式下流动相A(0.1%甲酸水溶液)和流动相B(含0.1%甲酸的甲醇-乙腈(1:1, v/v))。梯度洗脱程序:0.0~3.0 min, 40%B; 3.0~3.1 min, 40%B~60%B; 3.1~8.1 min, 60%B; 8.1~8.2 min, 60%B~85%B; 8.2~12.0 min, 85%B; 12.0~12.1 min, 85%B~98%B; 12.1~14.1 min, 98%B; 14.1~14.2 min, 98%B~40%B; 14.2~16.0 min, 40%B。ESI^-^模式下流动相A(纯水)和流动相B(乙腈)。梯度洗脱程序:0.0~1.0 min, 10%B; 1.0~1.1 min, 10%B~52%B; 1.1~3.5 min, 25%B; 3.5~3.6 min, 25%B~50%B; 3.6~8.2 min, 50%B~80%B; 8.2~8.3 min, 80%B~98%B; 8.3~10.2 min, 98%B; 10.2~10.3 min, 98%B~10%B; 10.3~12.0 min, 10%B。

离子源:电喷雾电离源正/负离子扫描模式;气帘气(氮气): 206 kPa;脱溶剂温度:500 ℃;离子源电压(正离子:5.0 kV,负离子:-4.5 kV);多反应监测(MRM)模式,具体参数详见表1。

### 1.3 样品预处理方法

称取样品2.0 g于离心管中,加入84%(v/v)乙腈水溶液20 mL涡旋振荡提取20 min,以8500 r/min离心3 min,移取上清液3 mL于另一离心管中,加入C_18_吸附剂140 mg及EMR-Lipid 25 mg涡旋混合净化3 min,结束后取出上清液2.0 mL氮吹浓缩至干,用1.0 mL初始流动相复溶后过0.22 μm聚四氟乙烯亲水性滤膜后进样分析,采用空白基质匹配曲线外标法定量。

### 1.4 确证方法

采用食品安全国家标准GB 5009.111-2016《食品中脱氧雪腐镰刀菌烯醇及其乙酰化衍生物的测定》第一法中通用型固相萃取柱对样品进行预处理。同时采用同位素内标法(包括^13^C_15_-脱氧雪腐镰刀菌烯醇作脱氧雪腐镰刀菌烯醇、^13^C_15_-雪腐镰刀菌烯醇作雪腐镰刀菌烯醇内标和^13^C_17_-3-乙酰化脱氧雪腐镰刀菌烯醇等)对其中脱氧雪腐镰刀菌烯醇及其衍生物进行定量分析。

## 2 结果与讨论

### 2.1 预处理方法的选择

不同种类的真菌毒素理化性质差异大,预处理方法除保持多组分高通量的同时还需要兼顾净化能力、分析速度等。本研究对6种常用的d-SPE净化剂(EMR-Lipid、C_18_、PSA、GCB、PCX和PAX)进行了考察。采用空白基质加标溶液(ESI^+^混合标准溶液加标量20 μL/20 mL提取液,ESI^-^混合标准溶液加标量为100 μL/20 mL提取液),按每3.0 mL空白基质加标溶液加入150 mg净化剂进行试验,结果以各真菌毒素的回收率表示。由表2可以发现,EMR-Lipid和C_18_对大部分毒素都有良好的回收率,EMR-Lipid在53%~104%范围,C_18_在79%~106%范围。但实验发现,使用C_18_净化剂处理的样品溶液经复溶后呈白色(小麦粉)和淡黄色(板栗粉)乳浊液状,而使用EMR-Lipid处理的样品溶液则十分澄清,表明EMR-L对脂类去除效果良好。同时也发现,EMR-Lipid净化剂对弱极性毒素的吸附作用明显大于C_18_,导致回收率低于C_18_,如恩链孢菌素、白僵菌素等(见表2)。为了兼顾样品溶液的净化效果和目标毒素的回收率,后续采用响应面试验设计法对两种净化剂用量进一步优化。

**表2:** 采用不同d-SPE净化剂时真菌毒素的回收率

Analyte	Recoveries/%						Analyte	Recoveries/%					
	EMR- Lipid	C_18_	PSA	GCB	PCX	PAX		EMR- Lipid	C_18_	PSA	GCB	PCX	PAX
Aflatoxin B_1_	88.3	91.3	94.3	0.3	63.3	84.3	Verruculogen	57.0	85.0	61.0	67.0	0.0	0.0
Aflatoxin B_2_	81.9	92.9	69.9	1.9	64.9	88.9	15-Acetyl-deoxynivalenol	93.7	95.7	114.7	96.7	74.7	96.7
Aflatoxin G_1_	87.0	96.0	77.0	1.0	64.0	76.0	3-Acetyl-deoxynivalenol	91.7	100.7	98.7	99.7	79.7	103.7
Aflatoxin G_2_	80.0	98.0	91.0	0.0	69.0	91.0	Alternariol monomethyl	68.7	83.7	6.7	0.7	79.7	111.7
Aflatoxin M_1_	87.1	102.1	19.1	1.1	83.1	80.1	ether						
Altenuene	56.1	96.1	0.1	0.1	41.1	13.1	α-Zearalanol	76.2	87.2	8.2	1.2	43.2	51.2
Beauvericin	52.9	88.9	24.9	0.1	0.1	0.0	α-Zearalenol	67.8	87.8	3.8	0.2	32.8	42.8
Chaetoglobosin A	78.3	95.3	0.3	0.3	8.3	0.3	Bongkrekic acid	63.0	85.0	0.0	65.0	78.0	5.0
Diacetoxyscirpenol	85.6	98.6	83.6	94.6	80.6	75.6	β-Zearalanol	75.0	95.0	8.0	2.0	42.0	51.0
Enniatin A	60.8	84.8	46.8	0.2	0.2	3.8	β-Zearalenol	69.8	91.8	6.8	0.8	31.8	42.8
Enniatin A_1_	59.2	87.2	71.2	0.2	0.2	4.2	Citreoviridin	86.7	93.7	85.7	44.7	78.7	80.7
Enniatin B	67.1	87.1	91.1	0.1	2.1	15.1	Deepoxy-deoxynivalenol	94.9	105.9	73.9	98.9	69.9	86.9
Enniatin B_1_	65.8	95.8	100.8	0.2	0.8	6.8	Deoxynivalenol	104.1	102.1	78.1	98.1	68.1	92.1
Gliotoxin	84.9	94.9	80.9	5.9	43.9	67.9	Fumagillin	73.9	86.9	0.1	0.1	64.9	42.9
HT-2	86.6	86.6	55.6	73.6	62.6	69.6	Fusarenone X	88.7	103.7	74.7	88.7	71.7	90.7
Neosolaniol	86.6	87.6	76.6	97.6	68.6	77.6	Nivalenol	103.3	103.3	21.3	87.3	46.3	64.3
Ochratoxin A	63.1	82.1	1.1	0.1	51.1	0.1	Patulin	92.1	101.1	6.1	78.1	65.1	81.1
Ochratoxin B	65.9	82.9	0.1	0.1	63.9	0.1	Penicillic acid	91.0	95.0	0.0	90.0	63.0	63.0
Sterigmatocystin	60.8	92.8	87.8	0.2	19.8	60.8	Penitrem A	54.3	89.3	42.3	0.3	71.3	82.3
T-2	82.3	79.3	90.3	75.3	60.3	82.3	Wortmannin	78.2	91.2	60.2	27.2	86.2	52.2
Tentoxin	95.8	84.8	79.8	26.8	45.8	72.8	Zearalanone	70.2	87.2	11.2	1.2	39.2	53.2
Toxoflavin	93.2	89.2	13.2	68.2	71.2	12.2	Zearalenone	63.6	84.6	6.6	0.4	35.6	47.6

### 2.2 响应面试验设计

在选择d-SPE净化剂种类后,以目标毒素回收率为响应值*Y*,以C_18_用量为变量*A*, EMR-Lipid用量为变量*B*。采用响应曲面-中心组合设计试验,设置5个不同水平的实验来减少实验中偶然误差的影响,其中*α*水平随变量数量而变化,当变量为2个时*α*为±1.41^[26]^。具体矩阵设计见表3,试验以随机次序进行,重复3次以平均值为结果(详见附表1, https://www.chrom-China.com),同时在中心点位(level 0)设置5次平行减少随机误差的影响。

**表3:** 中心组合设计试验矩阵

Factor	Symbol	Coded levels				
		-α (-1.41)	-1	0	1	+α (+1.41)
Dosage of	X_A_	58.6	100	200	300	341.4
C_18_ /mg						
Dosage of EMR-	X_B_	58.6	100	200	300	341.4
Lipid/mg						

根据上述矩阵进行试验后利用Design-expert 8.0.6.0软件对所得数据进行二次多元回归拟合,整理得到对实验因素一次项、交互项和二次项进行评估的回归方程:

$Y=\delta_{0}+\sum_{i=1}^{n} \delta_{i} X_{i}+\sum_{i=1}^{n} \delta_{i i} X_{i}^{2}+\sum_{i=1}^{n} \sum_{i=1}^{n} \delta_{i j} X_{i} X_{j}+\varepsilon$

*δ_ij_X_i_X_j_*+*ε*式中,*Y*为预测响应值,*X_i_*和*X_j_*代表独立变量,*δ*_0_为常数项,*δ_i_*为线性系数,*δ_ii_*为二次项系数,*δ_ij_*为交互项系数,*ε*为随机误差补偿项。以黄曲霉毒素B_1_为例,得到方程:

*Y*=90.69-0.39C_18_-5.11EMR+1.91C_18_×EMR+0.52 $C_{18}^{2}$ +0.84EMR^2^

然后对结果进行方差分析,通过拟合方程模型项、矢拟项、确定系数(*R*^2^)对生成的多项式模型质量进行评估。首先,所有模型项*P*值均为显著(<0.0132)、矢拟项为不显著(*P*值处于0.0809~0.9878范围),表明生成的二次多项式模型拟合度高,非常适用于当前条件下的数据预测;*R*^2^通常要求至少大于0.8表明理论预测与实际实验结果之间一致性良好^[27]^,而本实验中所有毒素的*R*^2^在0.8438~0.9974之间,表明生成的方程与实验数据拥有至少84.38%的符合度,对响应值的预测能力优秀,可信度较高^[28]^。最后分别对各变量求一阶偏导,计算出理论最佳实验条件为:140 mg C_18_及25 mg EMR-Lipid。在此条件下,真菌毒素预测回收率为79%~104%之间,随后进行了*n*=3的补充实验以验证预测准确性,结果显示(详见附表2, https://www.chrom-China.com),所有目标毒素实际回收率与理论预测值偏差小于15%,表明所建立模型的预测精度和准确度令人满意^[29]^。

### 2.3 典型色谱图

在1.2节色谱-质谱条件下,43种真菌毒素混合标准溶液的MRM图见图1。

**图1:**
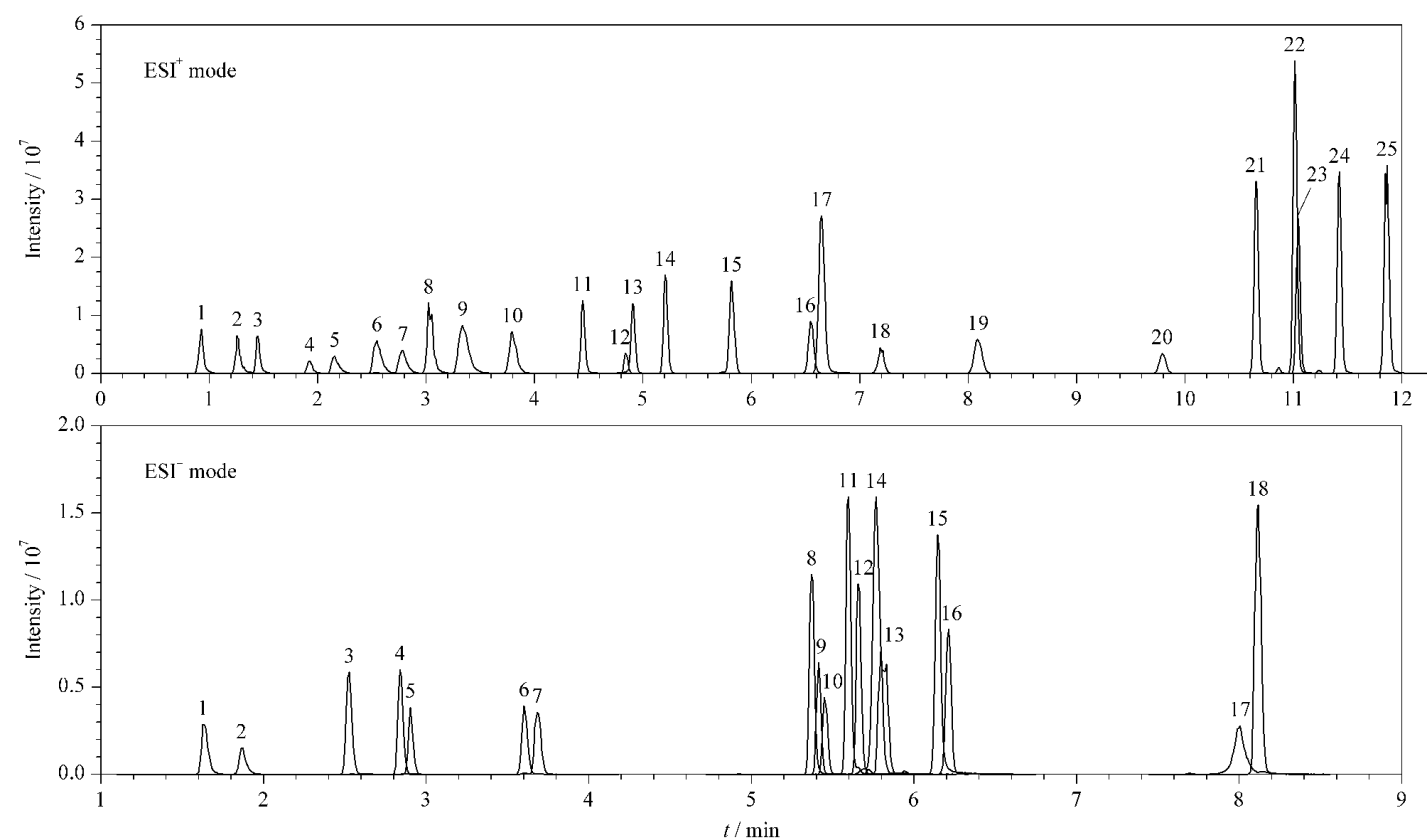
正/负离子模式下两个真菌毒素混合标准溶液的MRM图

### 2.4 方法回收率和精密度

在2.0 g小麦粉和板栗粉中,分别加入ESI^+^混合标准工作溶液2.0、20.0、100.0 μL,和ESI^-^标准工作溶液10.0、100.0、500.0 μL,配成低、中、高3个浓度的加标回收样品,然后按1.3节所述进行处理,按1.2节所述进行测定,重复测定6次。结果见表4,板栗粉中目标毒素的回收率在72.4%~109.4%之间(RSD≤7.5%),而在小麦粉基质中加标回收率在70.7%~112.9%之间(RSD≤9.3%)。

**表4:** 43种真菌毒素在板栗粉及小麦粉中3个水平下的加标回收率

Analyte	Chestnut flour				Wheat flour		
	Low level	Medium level	High level		Low level	Medium level	High level
Aflatoxin B_1_	89.5±5.5	91.0±3.5	99.7±5.1		89.8±2.9	90.0±7.9	97.7±6.8
Aflatoxin B_2_	87.2±3.4	91.2±4.9	99.6±5.3		98.7±2.8	93.8±3.1	93.2±1.1
Aflatoxin G_1_	86.7±3.2	91.8±2.6	96.1±2.1		91.8±5.3	93.2±2.4	96.4±4.0
Aflatoxin G_2_	93.8±4.7	95.6±2.7	104.8±1.6		93.3±4.2	94.1±5.5	96.7±3.7
Aflatoxin M_1_	89.3±2.5	98.9±4.3	100.0±2.6		79.8±2.9	96.8±4.3	95.8±2.8
Altenuene	79.3±4.9	76.4±2.8	77.1±3.0		78.3±6.9	79.7±4.4	82.7±2.9
Beauvericin	83.7±7.1	85.3±2.9	89.6±5.6		79.6±2.7	85.9±3.1	86.8±2.5
Chaetoglobosin A	87.0±3.0	81.9±3.2	90.6±1.7		90.4±5.3	91.8±3.5	90.7±3.8
Diacetoxyscirpenol	86.6±4.2	92.9±5.8	92.3±2.6		87.7±3.6	87.2±2.9	91.3±5.3
Enniatin A	75.8±6.3	81.1±5.1	80.4±2.0		72.5±8.6	83.4±5.1	88.6±4.7
Enniatin A_1_	77.1±6.1	74.5±5.5	75.8±2.4		75.3±5.8	76.6±5.1	78.1±4.1
Enniatin B	83.6±7.5	82.4±2.9	85.7±5.0		73.5±2.8	75.6±3.1	79.6±2.3
Enniatin B_1_	80.5±4.6	84.3±2.3	80.1±2.8		74.1±6.7	81.0±4.9	82.7±3.3
Gliotoxin	84.0±2.1	86.2±6.4	88.1±3.5		91.7±3.0	91.5±3.1	87.8±3.0
HT-2	87.4±6.7	90.7±5.4	102.2±5.0		89.2±6.1	87.3±3.5	86.4±5.3
Neosolaniol	87.0±4.5	89.9±2.8	93.6±2.2		84.1±2.7	86.1±3.9	94.1±2.8
Ochratoxin A	79.9±5.2	83.8±3.3	82.9±5.8		75.5±1.7	83.9±3.6	82.1±1.6
Ochratoxin B	84.1±3.9	86.0±3.5	82.9±3.3		78.3±2.9	81.8±4.2	79.2±3.2
Sterigmatocystin	74.8±6.5	78.7±3.6	78.0±3.9		79.5±4.3	75.6±3.8	79.4±2.5
T-2	86.9±4.6	83.6±4.1	87.7±6.3		85.5±4.6	84.3±2.9	89.4±2.0
Tentoxin	92.2±5.4	87.1±2.3	93.8±3.4		98.4±5.7	84.6±3.2	94.5±3.7
Toxoflavin	96.9±1.0	92.9±5.4	91.9±5.1		85.6±2.8	89.3±2.8	91.7±4.9
Verruculogen	83.6±3.7	82.5±2.2	84.4±3.5		88.3±3.3	85.5±2.6	90.7±5.0
15-Acetyl-deoxynivalenol	96.9±2.1	93.9±1.5	92.6±1.3		89.6±1.7	86.4±2.1	92.7±1.5
3-Acetyl-deoxynivalenol	86.9±1.7	100.1±0.8	90.0±1.0		87.5±2.5	96.4±1.7	89.1±1.2
Alternariol monomethyl ether	78.8±1.2	73.6±0.9	79.0±1.4		79.9±3.1	78.0±1.6	82.4±1.5
α-Zearalanol	82.5±2.2	87.1±2.6	83.6±1.4		87.5±5.6	85.5±1.6	87.2±1.7
α-Zearalenol	76.4±2.2	79.7±4.2	79.8±2.1		74.9±2.2	80.7±1.4	83.2±2.1
Bongkrekic acid	78.8±6.0	79.2±6.7	85.7±3.5		78.1±6.8	78.8±9.3	83.9±3.9
β-Zearalanol	83.6±2.1	87.6±2.1	87.4±1.3		87.5±3.1	85.2±1.3	79.8±1.2
β-Zearalenol	81.0±1.2	86.6±4.7	94.9±1.6		88.9±2.9	86.4±1.3	86.6±1.3
Citreoviridin	97.0±6.3	105.3±3.3	109.4±2.5		96.6±2.5	102.7±4.3	106.7±2.3
Deepoxy-deoxynivalenol	86.5±6.4	90.8±3.0	102.4±1.8		98.5±1.8	112.9±2.9	106.1±1.5
Deoxynivalenol	104.8±3.9	96.7±4.0	90.1±1.0		94.4±5.3	99.3±2.9	92.6±1.7
Fumagillin	80.1±4.1	80.9±5.4	81.0±3.8		85.3±5.3	90.2±4.7	90.4±2.9
Fusarenone X	86.9±3.8	86.3±3.7	91.3±3.1		87.4±4.1	99.4±8.3	89.8±3.7
Nivalenol	92.2±3.2	100.6±2.9	97.3±4.2		85.7±5.3	89.4±2.9	89.7±4.2
Patulin	96.6±3.9	82.4±2.0	89.9±1.1		87.2±3.7	83.8±3.6	87.9±2.3
Penicillic acid	90.1±1.9	100.6±2.2	92.8±1.8		88.8±1.3	93.4±1.5	90.6±1.7
Penitrem A	76.5±5.1	80.9±3.7	81.0±6.5		75.5±4.1	80.5±5.9	83.4±1.1
Wortmannin	79.4±2.5	82.1±3.2	81.9±1.3		75.0±1.0	89.1±2.6	92.4±1.9
Zearalanone	72.4±1.3	80.5±1.6	82.4±2.0		70.7±3.5	78.1±4.0	82.6±1.3
Zearalenone	74.5±1.8	82.7±2.8	88.5±1.5		82.3±3.2	91.8±1.7	89.3±1.0

### 2.5 方法的线性关系、基质效应和检出限

基质效应以基质匹配曲线斜率与溶剂标准曲线斜率的比值(即基质匹配曲线斜率/标准溶液曲线斜率)来计算。实验结果表明,在设定的浓度范围内小麦和板栗的基质匹配曲线线性关系良好,线性相关系数均大于0.9991。在小麦基质中有15种毒素受到较强抑制效应(<80%),为48%(毒黄素)~76%(恩链孢菌素B_1_),有4种毒素受到增强效应(>120%),最高为烟曲霉素(128%);在板栗基质中,共18种毒素受到抑制效应,为41%(恩链孢菌素A_1_)~74%(15-乙酰化-脱氧雪腐镰刀菌烯醇),增强效应中同样以烟曲霉素为最高(112%)。分别以10倍信噪比确定不同基质的定量限,最终两种基质中的定量限均处于0.1~20.0 μg/kg之间(见表5)。可以发现,在多毒素分析中部分毒素即使经过预处理净化仍然受到较强基质抑制效应,但依靠LC-MS较高的灵敏度仍然可以满足小麦粉及板栗粉中痕量毒素的分析要求。

**表5:** 43种真菌毒素在板栗粉及小麦粉中的基质效应及定量限

Analyte	LOQ/ (μg/kg)	Matrix effects/%		Analyte	LOQ/ (μg/kg)	Matrix effects/%	
		Chestnut flour	Wheat flour			Chestnut flour	Wheat flour
Aflatoxin B_1_	0.10	99.7	101.4	Verruculogen	0.50	41.0	48.8
Aflatoxin B_2_	0.10	100.0	99.1	15-Acetyl-deoxynivalenol	10.0	74.1	84.2
Aflatoxin G_1_	0.10	91.8	96.9	3-Acetyl-deoxynivalenol	10.0	69.7	102.7
Aflatoxin G_2_	0.10	70.5	73.9	Alternariol monomethyl ether	0.10	106.9	103.7
Aflatoxin M_1_	0.10	86.8	95.9	α-Zearalanol	0.50	93.0	125.3
Altenuene	4.0	70.0	68.7	α-Zearalenol	0.50	87.2	83.6
Beauvericin	0.20	108.1	81.4	Bongkrekic acid	20.0	63.9	48.8
Chaetoglobosin A	4.0	68.8	68.5	β-Zearalanol	0.50	88.4	86.2
Diacetoxyscirpenol	0.50	101.4	90.5	β-Zearalenol	0.50	75.9	49.4
Enniatin A	0.20	81.8	95.6	Citreoviridin	5.0	95.9	77.6
Enniatin A_1_	0.20	41.4	86.7	Deepoxy-deoxynivalenol	5.0	80.2	84.1
Enniatin B	0.20	70.4	73.6	Deoxynivalenol	10.0	89.8	99.0
Enniatin B_1_	0.20	72.8	75.7	Fumagillin	5.0	111.6	128.4
Gliotoxin	5.0	103.6	80.8	Fusarenone X	5.0	61.3	83.6
HT-2	10.0	73.5	69.6	Nivalenol	4.0	70.5	88.0
Neosolaniol	1.0	93.4	86.6	Patulin	5.0	75.2	79.8
Ochratoxin A	0.10	96.9	98.8	Penicillic acid	1.0	104.3	83.6
Ochratoxin B	0.10	99.2	120.1	Penitrem A	0.50	79.6	58.4
Sterigmatocystin	0.10	96.4	97.3	Wortmannin	5.0	70.5	63.4
T-2	0.40	70.3	67.7	Zearalanone	0.50	89.1	83.5
Tentoxin	0.40	93.1	122.4	Zearalenone	0.50	87.8	84.7
Toxoflavin	1.0	70.5	48.2				

### 2.6 测定结果及分析

对实际样品进行预处理后分析,分别采用基质匹配曲线外标法完成定量,测定结果见表6。

**表6:** 样品中阳性毒素及其检出浓度范围

Positive mycotoxin	Chestnut flour (n=48)			Wheat flour (n=80)	
	Positive quantity (incidence)	Content range (mean)/(μg/kg)		Positive quantity (incidence)	Content range (mean)/(μg/kg)
Aflatoxin B_1_	21 (43.8%)	0.1-8.3 (1.6)		12 (15.0%)	0.1-5.2 (1.0)	
Aflatoxin B_2_	20 (41.7%)	0.1-3.7 (1.0)		10 (12.5%)	0.1-1.2 (0.4)	
Aflatoxin G_1_	12 (25.0%)	0.2-5.9 (2.3)		4 (5.0%)	0.1-0.5 (0.2)	
Aflatoxin G_2_	12 (25.0%)	0.1-2.5 (1.2)		3 (3.8%)	0.1-0.2 (0.2)	
Alternariol monomethyl ether	30 (62.5%)	0.1-5.1 (1.5)		62 (77.5%)	0.2-12.6 (2.1)	
Beauvericin	43 (89.6%)	0.4-50.3 (14.2)		64 (80.0%)	0.3-67.7 (24.6)	
Chaetoglobosin A	10 (20.8%)	4.5-35.8 (14.2)		N. D.	N. D.	
Diacetoxyscirpenol	11 (22.9%)	0.6-6.5 (2.3)		13 (16.2%)	0.5-9.6 (2.9)	
Enniatin A	27 (56.2%)	0.6-131.4 (21.5)		53 (66.2%)	0.7-259.3 (29.3)	
Enniatin A_1_	33 (68.8%)	0.5-206.0 (25.8)		58 (72.5%)	5.3-406.4 (42.1)	
Enniatin B	33 (68.8%)	2.2-373.4 (57.5)		70 (87.5%)	4.5-822.0 (71.2)	
Enniatin B_1_	30 (62.5%)	4.4-364.7 (42.6)		66 (82.5%)	2.4-587.7 (55.4)	
Ochratoxin A	16 (33.3%)	0.4-24.7 (5.9)		18 (22.5%)	0.12-5.6 (1.6)	
Ochratoxin B	6 (12.5%)	0.6-4.8 (3.0)		N. D.	N. D.	
Penicillic acid	1 (2.1%)	1.9		N. D.	N. D.	
Tentoxin	33 (68.8%)	0.6-33.5 (8.5)		58 (72.5%)	0.1-30.2 (7.6)	
Deoxynivalenol	N. D.	N. D.		77 (96.2%)	14.3-2123.6 (297.1)	
3-Acetyl-deoxynivalenol	N. D.	N. D.		8 (10.0%)	12.1-85.6 (32.2)	
15-Acetyl-deoxynivalenol	N. D.	N. D.		N. D.	N. D.	
Nivalenol	N. D.	N. D.		12 (15.0%)	14.6-64.7 (29.2)	
Tentoxin	30 (62.5%)	0.2-9.5 (1.8)		43 (53.8%)	0.16-24.0 (3.6)	

结果表明,两种基质中均检出17种真菌毒素,经对比分析发现:4种黄曲霉毒素在板栗粉中超标率(黄曲霉毒素B_1_>2.0 μg/kg,或黄曲霉毒素总量>4.0 μg/kg)高于小麦粉,但总体阳性率不高(<45%),不具备作为标志物的特性;交链孢酚单甲醚、腾毒素、白僵菌素、恩链孢菌素及玉米赤霉烯酮在两类食物中检出率较高,但在阳性率和污染浓度之间仍缺少显著性差异;球毛壳菌素A、青霉酸、赭曲霉毒素B 3种毒素仅在板栗粉中被检出,但三者最高的阳性率也仅为20.8%,作为特异性标志物代表性不足。值得关注的是两类食物中的脱氧雪腐镰刀菌烯醇,其在板栗粉中均未检出(检出限为10.0 μg/kg),而小麦粉中检出率达96.2%,平均浓度297.1 μg/kg。另外,实验室自制的10份霉变板栗粉样品除赭曲霉毒素A、黄曲霉毒素B_1_、B_2_含量略微增加0.7~4.1 μg/kg外,其余毒素浓度变化均不显著,脱氧雪腐镰刀菌烯醇仍然未产生。作者认为,上述现象产生的主要原因在于板栗粉和小麦粉中常见产毒菌种的差异。镰刀菌是小麦中最常见的菌种之一,当作物在田间时就可能受到镰刀菌的侵染,而脱氧雪腐镰刀菌烯醇及其衍生物主要是由禾谷镰刀菌(*F. graminearum*)和黄色镰刀菌(*F. culmorum*)等产生^[30]^。另一方面,从板栗制品中分离出来的菌种主要包括曲霉属(*Aspergillus*)、青霉属(*Penicillium*)和链格孢霉属(*Alternaria*)^[14,15]^,但目前无相关证据证明镰刀菌属为板栗基质的主要污染菌种。

因此,将脱氧雪腐镰刀菌烯醇作为区分小麦粉和板栗粉的标志物具有重要的参考价值。同时,脱氧雪腐镰刀菌烯醇的4种衍生物,包括3-乙酰化-脱氧雪腐镰刀菌烯醇、15-乙酰化-脱氧雪腐镰刀菌烯醇、雪腐镰刀菌烯醇、去环氧-脱氧雪腐镰刀菌烯醇,虽然实际检出率及污染并不显著,但是其作为脱氧雪腐镰刀菌烯醇的前体或衍生物,出现必然会伴随着脱氧雪腐镰刀菌烯醇,因此这4类毒素的出现也可补充作为判断的重要依据。

### 2.7 掺假标准验证及灵敏度测试

使用食品安全国家标准方法GB 5009.111-2016对所有板栗粉(包括10份自制霉变板栗粉)中的脱氧雪腐镰刀菌烯醇及其乙酰化衍生物进行确证分析,分析结果与本方法完全一致。

选择典型污染小麦粉(脱氧雪腐镰刀菌烯醇含量为282.4 μg/kg)和空白板栗粉,按污染小麦粉与板栗粉的质量比为5%/95%、10%/90%、20%/80%、50%/50%、75%/25%混合,按1.3节处理后进样分析。结果显示即使以最低5%质量的小麦粉掺假,在上述实验条件下同样可以在模拟掺假板栗粉中检出脱氧雪腐镰刀菌烯醇,表明方法的灵敏度足够用于判断掺假。

## 3 结论

本文建立的板栗粉和小麦粉中43种真菌毒素的d-SPE-UFLC-MS/MS法具有方法简便、快速、灵敏、准确的特点,可有效满足板栗粉和小麦粉中真菌毒素残留的检测要求。检测结果表明,脱氧雪腐镰刀菌烯醇及其4种衍生物可以作为两种食品的掺假标志物。
